# International evaluation of an artificial intelligence–powered electrocardiogram model detecting acute coronary occlusion myocardial infarction

**DOI:** 10.1093/ehjdh/ztad074

**Published:** 2023-11-28

**Authors:** Robert Herman, Harvey Pendell Meyers, Stephen W Smith, Dario T Bertolone, Attilio Leone, Konstantinos Bermpeis, Michele M Viscusi, Marta Belmonte, Anthony Demolder, Vladimir Boza, Boris Vavrik, Viera Kresnakova, Andrej Iring, Michal Martonak, Jakub Bahyl, Timea Kisova, Dan Schelfaut, Marc Vanderheyden, Leor Perl, Emre K Aslanger, Robert Hatala, Wojtek Wojakowski, Jozef Bartunek, Emanuele Barbato

**Affiliations:** Department of Advanced Biomedical Sciences, University of Naples Federico II, C.so Umberto I, 40, 80138 Naples, Italy; Cardiovascular Centre Aalst, OLV Hospital, Moorselbaan 164, Aalst 9300, Belgium; Powerful Medical, Bratislavska 81/37, 931 01 Samorin, Slovakia; Department of Emergency Medicine, Carolinas Medical Center, Charlotte, NC, USA; Department of Emergency Medicine, University of Minnesota, Minneapolis, MN, USA; Department of Emergency Medicine, Hennepin Healthcare, Minneapolis, MN, USA; Department of Advanced Biomedical Sciences, University of Naples Federico II, C.so Umberto I, 40, 80138 Naples, Italy; Cardiovascular Centre Aalst, OLV Hospital, Moorselbaan 164, Aalst 9300, Belgium; Department of Advanced Biomedical Sciences, University of Naples Federico II, C.so Umberto I, 40, 80138 Naples, Italy; Cardiovascular Centre Aalst, OLV Hospital, Moorselbaan 164, Aalst 9300, Belgium; Department of Advanced Biomedical Sciences, University of Naples Federico II, C.so Umberto I, 40, 80138 Naples, Italy; Cardiovascular Centre Aalst, OLV Hospital, Moorselbaan 164, Aalst 9300, Belgium; Department of Advanced Biomedical Sciences, University of Naples Federico II, C.so Umberto I, 40, 80138 Naples, Italy; Cardiovascular Centre Aalst, OLV Hospital, Moorselbaan 164, Aalst 9300, Belgium; Department of Advanced Biomedical Sciences, University of Naples Federico II, C.so Umberto I, 40, 80138 Naples, Italy; Cardiovascular Centre Aalst, OLV Hospital, Moorselbaan 164, Aalst 9300, Belgium; Powerful Medical, Bratislavska 81/37, 931 01 Samorin, Slovakia; Powerful Medical, Bratislavska 81/37, 931 01 Samorin, Slovakia; Faculty of Mathematics, Physics and Informatics, Comenius University in Bratislava, Bratislava, Slovakia; Powerful Medical, Bratislavska 81/37, 931 01 Samorin, Slovakia; Powerful Medical, Bratislavska 81/37, 931 01 Samorin, Slovakia; Department of Cybernetics and Artificial Intelligence, Technical University of Kosice, Kosice, Slovakia; Powerful Medical, Bratislavska 81/37, 931 01 Samorin, Slovakia; Powerful Medical, Bratislavska 81/37, 931 01 Samorin, Slovakia; Powerful Medical, Bratislavska 81/37, 931 01 Samorin, Slovakia; Powerful Medical, Bratislavska 81/37, 931 01 Samorin, Slovakia; Faculty of Medicine and Dentistry, Barts and The London School of Medicine and Dentistry, London, UK; Cardiovascular Centre Aalst, OLV Hospital, Moorselbaan 164, Aalst 9300, Belgium; Cardiovascular Centre Aalst, OLV Hospital, Moorselbaan 164, Aalst 9300, Belgium; Department of Cardiology, Rabin Medical Center, Petah Tikvah, Israel; Department of Cardiology, Basaksehir Cam and Sakura City Hospital, Istanbul, Turkey; Department of Arrhythmia and Pacing, National Institute of Cardiovascular Diseases, Bratislava, Slovakia; Department of Cardiology and Structural Heart Diseases, Medical University of Silesia, Katowice, Poland; Cardiovascular Centre Aalst, OLV Hospital, Moorselbaan 164, Aalst 9300, Belgium; Department of Clinical and Molecular Medicine, Faculty of Medicine and Psychology, Sapienza University of Rome, Rome, Italy

**Keywords:** Electrocardiogram, Artificial intelligence, Acute coronary syndrome, Myocardial infarction, Occlusion myocardial infarction, NSTEMI

## Abstract

**Aims:**

A majority of acute coronary syndromes (ACS) present without typical ST elevation. One-third of non–ST-elevation myocardial infarction (NSTEMI) patients have an acutely occluded culprit coronary artery [occlusion myocardial infarction (OMI)], leading to poor outcomes due to delayed identification and invasive management. In this study, we sought to develop a versatile artificial intelligence (AI) model detecting acute OMI on single-standard 12-lead electrocardiograms (ECGs) and compare its performance with existing state-of-the-art diagnostic criteria.

**Methods and results:**

An AI model was developed using 18 616 ECGs from 10 543 patients with suspected ACS from an international database with clinically validated outcomes. The model was evaluated in an international cohort and compared with STEMI criteria and ECG experts in detecting OMI. The primary outcome of OMI was an acutely occluded or flow-limiting culprit artery requiring emergent revascularization. In the overall test set of 3254 ECGs from 2222 patients (age 62 ± 14 years, 67% males, 21.6% OMI), the AI model achieved an area under the curve of 0.938 [95% confidence interval (CI): 0.924–0.951] in identifying the primary OMI outcome, with superior performance [accuracy 90.9% (95% CI: 89.7–92.0), sensitivity 80.6% (95% CI: 76.8–84.0), and specificity 93.7 (95% CI: 92.6–94.8)] compared with STEMI criteria [accuracy 83.6% (95% CI: 82.1–85.1), sensitivity 32.5% (95% CI: 28.4–36.6), and specificity 97.7% (95% CI: 97.0–98.3)] and with similar performance compared with ECG experts [accuracy 90.8% (95% CI: 89.5–91.9), sensitivity 73.0% (95% CI: 68.7–77.0), and specificity 95.7% (95% CI: 94.7–96.6)].

**Conclusion:**

The present novel ECG AI model demonstrates superior accuracy to detect acute OMI when compared with STEMI criteria. This suggests its potential to improve ACS triage, ensuring appropriate and timely referral for immediate revascularization.

## Structured Graphical Abstract

**Structured Graphical Abstract:**
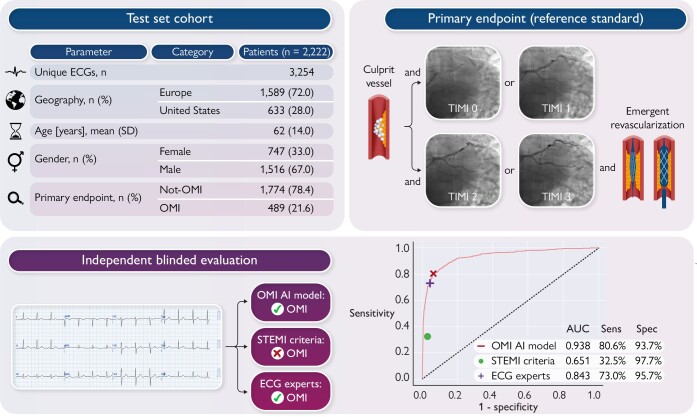

## Introduction

Patients with an acutely occluded or obstructive culprit coronary artery (acute coronary occlusion myocardial infarction, abbreviated as ‘OMI’), who will benefit from emergent reperfusion therapy, are currently identified on the basis of electrocardiographic ST-segment elevation [ST-elevation myocardial infarction (STEMI)].^1,2^ However, the pathophysiology of acute coronary syndrome (ACS) due to thrombotic occlusive coronary stenosis is often dynamic and may impact electrocardiogram (ECG) appearance at the time of the first patient contact. Accordingly, growing evidence suggests that the current ACS classification dichotomizing patients as STEMI or non-STEMI (NSTEMI) is unsatisfactory for the timely diagnosis of OMI, as also recognized by the 2022 American College of Cardiology Chest Pain Expert Consensus.^3^ On the one hand, 25–30% of NSTEMI patients present with acute coronary occlusion with insufficient collateral circulation as discovered only on delayed coronary angiography (CAG).^4^ The delayed invasive management in these patients is associated with two-fold higher short-term and long-term mortality.^4,5^ On the other hand, 15–25% of catheterization laboratory activations due to suspected STEMI eventually reveal no culprit lesions or a non-ischaemic aetiology of ST elevation (STE).^6–8^ A plethora of ECG criteria have been proposed to increase diagnostic sensitivity for OMI compared with the current guideline–based STEMI criteria and to differentiate OMI from mimics.^3,5,9–15^ Yet, their adoption is limited due to their complexity and unclear inter-evaluator reliability.

Recently, a machine learning approach has outperformed standard ECG criteria in detecting acute OMI correlating 73 hand-selected morphological ECG features and clinical parameters.^16^ In this study, we introduce an international validation of an automated deep learning artificial intelligence (AI) model detecting acute OMI using only a single-standard 12-lead ECG as input and hypothesize that it would outperform the existing state-of-the-art ECG criteria for the detection of acute OMI and match the performance of interpreters with special expertise in ECG OMI diagnosis.

## Methods

### Study design

This is a retrospective study following four key stages: (i) the development of an OMI AI model for the detection of acute OMI using only single-standard 12-lead ECGs as input (‘derivation cohort’); (ii) the evaluation of a blinded AI model in a geographically distinct test set spanning Europe and USA; (iii) the comparison of an AI model with the existing state-of-the-art criteria detecting OMI using 12-lead ECGs; and (iv) the performance analysis of an AI model in subgroups. Each of these steps is described below. This retrospective study was approved by the local ethics committee for human research and complied with the Declaration of Helsinki.

### Data sources and processing

**Figure 1:**
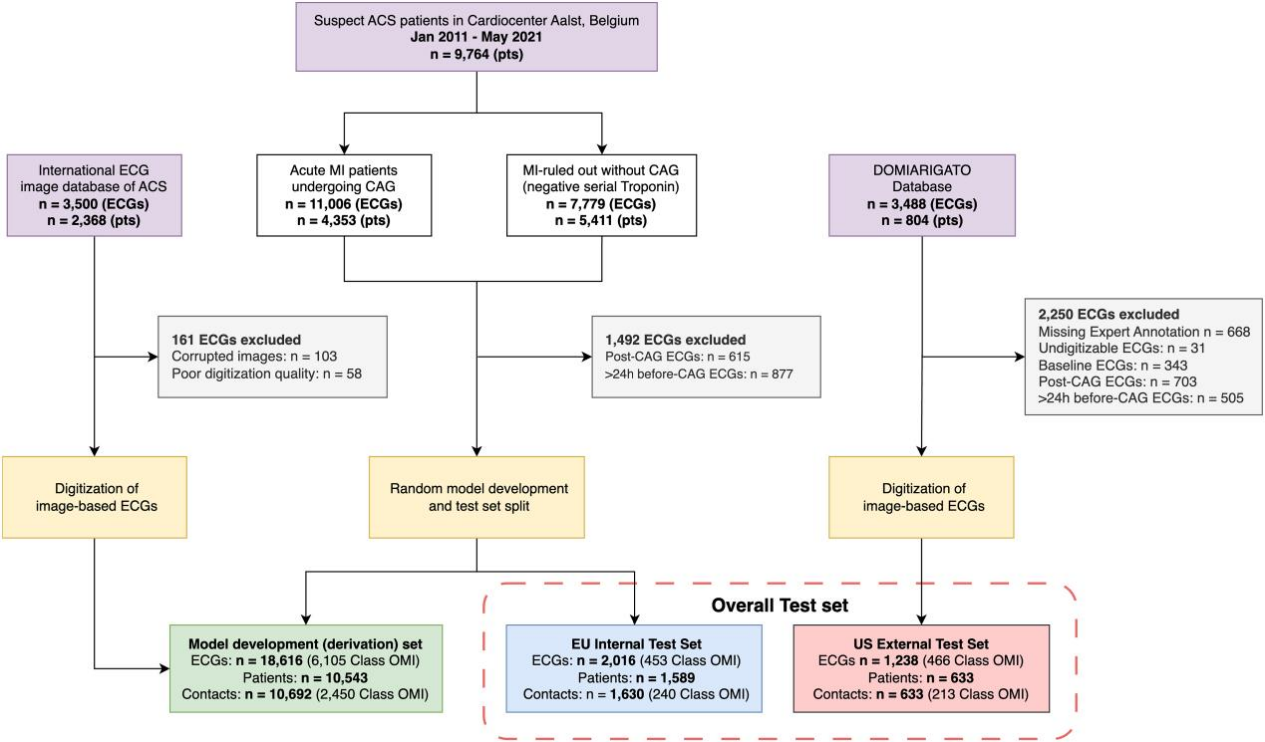
A PRISMA flow chart showing data sources and study populations. Suspect acute coronary syndrome patients identified, exclusions (in grey), and the final study population split into a model development set (in green), EU internal test set (in blue), and US external test set (in red). ECG, electrocardiogram; ACS, acute coronary syndrome; pts, patients; CAG, coronary angiography; MI, myocardial infarction; OMI, occlusion myocardial infarction.

Clinical data from 9764 patients who presented with suspected ACS to the Cardiovascular Centre Aalst in Belgium during the period between 2011 and 2021 and a clinically validated international image database of 2368 ACS patients (see Supplementary material online for a detailed description) were considered for the AI model development and testing. Waveform data, sampled at 500 Hz, were exported from the MUSE ECG data management system (GE Healthcare, Chicago, IL, USA) in XML format. The images of ECG tracings from multiple device vendors within the international image database of ACS patients were converted to digital waveforms using Ce-certified PMcardio ECG digitization technology (Powerful Medical, Samorin, Slovakia). Electrocardiograms recorded >24 h before CAG and post-CAG or ECGs with poor signal quality were discarded. The patients retained upon exclusions were randomly split into a model development (derivation) set and an internal Europe (EU) testing data set, ensuring that patients with more than one (recurrent) ACS contact were present in only one of the sets. Time from the first ECG to intervention was recorded for all cases if the patients underwent coronary angiography. The derivation set included ECGs adjudicated as OMI or not OMI by interpreters with special expertise in ECG OMI diagnosis (S.W.S. and H.P.M.) and by clinically validated angiographic outcome data (see details below under ‘Occlusion myocardial infarction artificial intelligence model development’). ‘Not OMI’ encompasses patients who either do not have acute myocardial infarction (MI) or have acute non-occlusion MI (non-OMI or NOMI) with either no culprit vessel identified angiographically or where the identified culprit vessel does not require immediate revascularization. A full overview of the data sources and inclusions and exclusions is available in *Figure 1*.

### Primary and secondary outcomes

The primary outcome was the AI model’s ability to identify patients with angiographically confirmed OMI using only single-standard 12-lead ECGs. The primary definition of OMI was modelled from previous studies^2,5,9,10,17–19^ and consisted of clinical symptoms and a troponin elevation consistent with the fourth universal definition of MI^20^ and angiographic evidence of acute culprit coronary stenosis with either (i) a thrombolysis in myocardial infarction (TIMI) flow grade of 0–1 or (ii) a TIMI flow grade of 2–3 with emergent or urgent percutaneous revascularization. Patients without any dynamic changes detected in serial biomarker testing were safely ruled out for OMI regardless of undergoing coronary angiography. This outcome was considered the reference standard for all analyses unless otherwise specified.

Secondary outcomes included the following: (i) OMI AI model performance across demographic and electrocardiographic subgroups; (ii) a comparison of the AI model performance against the existing criteria for detecting acute coronary occlusion from 12-lead ECGs,^9,20^ (iii) a sensitivity analysis of AI model performance using different angiographic and laboratory cut-offs of OMI, and (iv) an analysis of misclassified cases.

### Occlusion myocardial infarction artificial intelligence model development

Digital and digitized 12-lead ECG input data collected from sources described above were standardized into a 3 × 4 ECG format (2.5 s per lead). For longer ECG formats, the first 2.5 s of limb leads and the last 2.5 s of pre-cordial leads were used. The model development set was further subdivided into a training set and a validation set. A deep convolutional neural network architecture was deployed in model development and included two key components: feature extraction and classification. The feature-extraction component, comprised of two convolutional layers and six residual blocks (∼60 000 parameters), was designed to extract features in a lead-specific manner. The second classification component combined all extracted features and processed them through two fully connected layers (∼150 000 parameters). An analysis of each lead, and an integration of the knowledge gained, mimic the analytical approach of human experts to make a final diagnosis. Artificial intelligence model explainability is described in the Supplementary material online. The validation data set was used for hyperparameter tuning and threshold selection. The optimal model threshold was selected by maximizing Matthew’s correlation coefficient (MCC). An additional threshold was selected to match the specificity of STEMI criteria.

### EU internal testing data set

Independent clinical reviewers adjudicated the angiographic data of all patients included in the EU internal testing data set. The process of clinical verification included the blinded identification of culprit vessels, their visual assessment of coronary stenosis, TIMI flow, the presence of sufficient collateral flow on all individual angiograms, and the documentation of treatment strategy. If applicable, revascularization time, defined as the duration between the first ECG and the time when a balloon was inflated or when the wire crossed the lesion, was documented.

### US external testing data set

Electrocardiogram and outcome data from the Diagnosis of Occlusion MI And Reperfusion by Interpretation of the electrocardioGram in Acute Thrombotic Occlusion (DOMI ARIGATO) database (clinical trials.gov number NCT03863327) were included in the US external testing cohort. Data collection and processing of this database are explained elsewhere.^2^ Briefly, the DOMI ARIGATO database collected ECGs, laboratory data, and the clinically verified angiograms of patients presenting with ACS at two US sites, Stony Brook University Hospital and Hennepin County Medical Center. Electrocardiograms were interpreted and manually annotated by ECG experts blinded to all clinical data other than age and sex. Baseline ECGs, post-CAG ECGs, and ECGs with missing expert annotations were removed from the testing cohort.

### Benchmarking

The performance of the developed AI model was evaluated by comparing it with blinded physician annotations of electrocardiographic ‘STEMI criteria’ as a surrogate indicator of OMI, as well as subjective ECG expert annotations of OMI referred to as ‘ECG Experts’. The presence of STEMI criteria was assessed based on the fourth Universal Definition of Myocardial Infarction and included new STE ≥1 mm in two contiguous leads other than leads V2 and V3 (where STE ≥2 mm in men ≥40 years, ≥2.5 mm in men <40 years, and ≥1.5 mm in women).^20^ Two ECG experts (S.W.S. and H.P.M.) with expertise in OMI detection (94% agreement, kappa = 0.849) annotated all tracings for the presence of OMI, blinded to all clinical information.^9^ All ECGs in the overall testing data set were independently labelled using the two methods described in this paragraph. In patients with multiple ECGs prior to coronary angiography, a maximum interpretation per patient was retained for the benchmarking. The time to diagnose OMI was noted for each criterion by measuring the duration from the patient’s initial ECG to the accurate identification of OMI on subsequent ECGs. In cases where the criteria were unable to detect OMI in any ECG before CAG, the time to diagnosis was considered equivalent to the time to CAG.

### Statistical analyses

Statistical analysis was performed using Python programming language and the following open-source libraries: *tableone*, *lifelines*, and *pandas*. Continuous statistics with normal distribution were expressed as mean ± standard deviation and compared by using Student’s *t*-tests. Continuous variables with a non-normal distribution were presented as median with inter-quartile ranges (IQRs) and reached by the Mann–Whitney *U* test.^21^ If appropriate, categorical variables were reported by frequencies and percentages and compared with the *χ*^2^ test and Fisher’s exact test. The performances of the OMI AI model, ECG experts, and STEMI criteria were evaluated using the following standard evaluation metrics: sensitivity, specificity, accuracy, negative predictive value, positive predictive value, MCC, and area under the curve (AUC). For all evaluation metrics, we estimated the confidence intervals (CIs) at 95% by 10 000 iterations of the bootstrap method.^22^ In the subgroup analysis, patients’ ECGs were stratified according to ECG measurement (QRS duration and heart rate) and ECG diagnostic annotations (rhythm, ventricular hypertrophy, bundle branch blocks).

## Results

### Derivation set characteristics

A total of 18 616 ECGs from 10 543 patients (age 66 ± 14 years, 65.9% males, 22.9% OMI) with clinically validated outcomes originating from the Cardiovascular Centre Aalst and an international image database of ACS patients were included in the AI model development. The sample characteristics are shown in *Table 1*.

**Table 1:** Sample characteristics of the model development and EU and US test sets

Parameter	Cat.	Model development set	Overall test set	*P*-value (all)	Internal EU test set	External US test set	*P*-value (overall test sets)
Unique patients, *n*		10 543	2222		1589	633	
Unique ECGs, *n*		18 616	3254		2016	1238	
Age (years), mean (SD)		66 (14.0)	62 (14.0)	**<0**.**001**	63 (14.0)	61 (14.0)	**<0**.**001**
Gender, *n* (%)	Female	3394 (34.1)	747 (33.0)	0.336	543 (33.3)	204 (32.2)	0.658
	Male	6560 (65.9)	1516 (67.0)	0.336	1087 (66.7)	429 (67.8)	0.658
Unique contacts, *n*		10 692	2263		1630	633	
Primary outcome, *n* (%)	Class non-OMI	8242 (77.1)	1774 (78.4)	0.187	1370 (84.0)	404 (63.8)	**<0**.**001**
	Class OMI	2450 (22.9)	489 (21.6)	0.187	260 (16.0)	229 (36.2)	**<0**.**001**

Values in bold indicate statistically significant differences (*p* < 0.05). Cat., category; SD, standard deviation; OMI, occlusion myocardial infarction; ECG, electrocardiogram.

### Test set characteristics

The procedural characteristics of both testing cohorts are given in *Table 2*. The overall test set included 3254 ECGs from 2222 patients (age 62 ± 14 years, 67% males, 21.6% OMI). Of these, 2016 ECGs from 1630 contacts [with 240 (16%) OMI] were from the internal EU testing cohort, and 1238 ECGs from 633 contacts [with 213 (36.2%) OMI] were from the US testing cohort. The prevalence of OMI differed between the internal EU and the external US test sets, 16% compared with 36.2%, respectively (*P* < 0.001). The contacts included in the US test set were younger, had more ECGs recorded before catheterization, and were more likely to present with a STEMI-positive ECG. Gender, peak troponin, and the TIMI flow of culprit vessels did not differ significantly between the two cohorts.

**Table 2:** Procedural characteristics of the patient contacts in the EU and US test sets

Parameter	Cat.	Overall test sets	Internal EU test set	External US test set	*P*-value
ECG presentation, *n* (%)	STEMI	186 (8.2)	76 (4.7)	110 (17.4)	**<0**.**001**
	Non-STEMI	2077 (91.8)	1554 (95.3)	523 (82.6)	**<0**.**001**
Average ECGs per patient, mean (SD)		1.4 (0.9)	1.2 (0.6)	2.0 (1.2)	**<0**.**001**
Admission troponin T (ng/L), median (Q1, Q3)		7.4 (4.2, 13.5)	7.4 (4.2, 13.5)	NA	NA
Peak troponin T (ng/L), median (Q1, Q3)		31.8 (5.0, 1457.2)	11.8 (4.3, 340.5)	340.0 (11.0, 2820.1)	**<0**.**001**
CAG performed, *n* (%)		1408 (62.2)	948 (58.2)	460 (72.7)	**<0**.**001**
Time to CAG (h), median (Q1, Q3)		13.4 (2.5, 19.6)	17.3 (4.9, 20.4)	3.8 (0.8, 15.1)	**<0**.**001**
Time to CAG, *n* (%)	Late (12–24 h)	715 (50.9)	576 (61.0)	139 (30.2)	**<0**.**001**
	Delayed (4–12 h)	256 (18.2)	167 (17.7)	89 (19.3)	**<0**.**001**
	Early (2–4 h)	123 (8.8)	72 (7.6)	51 (11.1)	**<0**.**001**
	Immediate (<2 h)	310 (22.1)	129 (13.7)	181 (39.3)	**<0**.**001**
Culprit vessel, *n* (%)	None	1632 (72.1)	1316 (80.7)	316 (49.9)	**<0**.**001**
	Native	605 (26.7)	298 (18.3)	307 (48.5)	**<0**.**001**
	Graft	26 (1.1)	16 (1.0)	10 (1.6)	**<0**.**001**
Culprit artery, *n* (%)	LMCA	11 (1.7)	6 (1.9)	5 (1.6)	**<0**.**001**
	LAD	234 (37.1)	113 (36.0)	121 (38.2)	**<0**.**001**
	LCx	142 (22.5)	59 (18.8)	83 (26.2)	**<0**.**001**
	RCA	220 (34.9)	130 (41.4)	90 (28.4)	**<0**.**001**
	PDA	9 (1.4)	0 (0.0)	9 (2.8)	**<0**.**001**
	RI	11 (1.7)	2 (0.6)	9 (2.8)	**<0**.**001**
	Multi-vessel	4 (0.6)	4 (1.3)	0 (0.0)	**<0**.**001**
Culprit stenosis (%), median (Q1, Q3)		90.0 (70.0, 100.0)	80.0 (60.0, 100.0)	95.0 (90.0, 100.0)	**<0**.**001**
Culprit TIMI flow, *n* (%)	TIMI-0	244 (38.6)	119 (37.8)	125 (39.4)	0.911
	TIMI-1	38 (6.0)	19 (6.0)	19 (6.0)	0.911
	TIMI-2	70 (11.1)	33 (10.5)	37 (11.7)	0.911
	TIMI-3	279 (44.2)	143 (45.5)	136 (42.9)	0.911
Collateral flow, *n* (%)	None	284 (90.4)	284 (90.4)	NA	NA
	Mild	16 (5.1)	16 (5.1)	NA	NA
	Moderate	12 (3.8)	12 (3.8)	NA	NA
	High	2 (0.6)	2 (0.6)	NA	NA
Time to revascularization (h), median (Q1, Q3)		7.5 (2.1, 19.3)	7.5 (2.1, 19.3)	NA	NA
Treatment, *n* (%)	Conservative	706 (50.1)	525 (55.4)	181 (39.3)	**<0**.**001**
	PCI	699 (49.6)	422 (44.5)	277 (60.2)	**<0**.**001**

Values in bold indicate statistically significant differences (*p* < 0.05). Cat., category; CAG, coronary angiography; ECG, electrocardiogram; STEMI, ST-elevation myocardial infarction; SD, standard deviation; LMCA, left main coronary artery; LAD, left anterior descending artery; LCx, left circumflex artery; NA, not available; RCA, right coronary artery; PDA, posterior descending artery; RI, ramus interventricularis; TIMI, Thrombolysis in myocardial infarction; PCI, percutaneous coronary intervention.

### Artificial intelligence model performance

**Figure 2:**
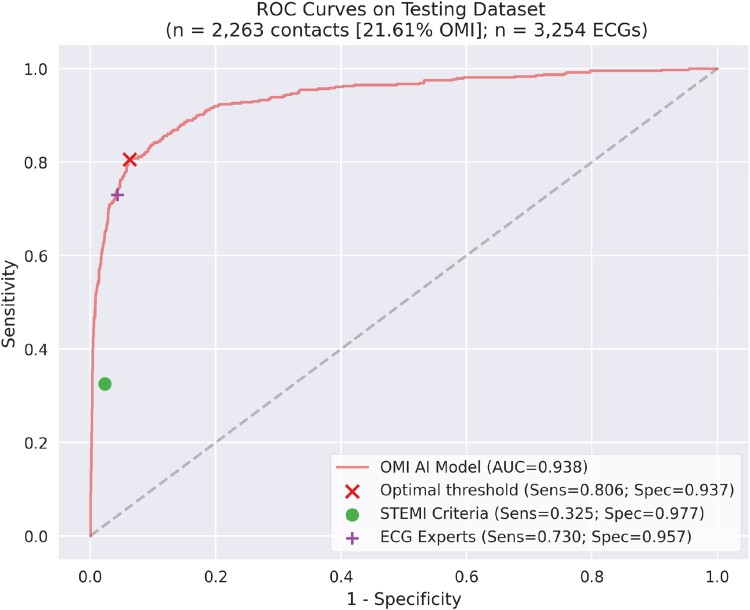
Artificial intelligence model performance on the overall testing data set. The receiver operating characteristic curve of the occlusion myocardial infarction artificial intelligence model (red) and the sensitivity and specificity of the occlusion myocardial infarction artificial intelligence model optimal threshold (red X), STEMI criteria (green dot), and electrocardiogram experts (purple cross) on combined EU and US testing cohorts. The AUC is 0.938 [*n* = 2263 contacts (21.61% occlusion myocardial infarction)]. OMI, occlusion myocardial infarction; AI, artificial intelligence; STEMI, ST-elevation myocardial infarction.

The OMI AI model with an optimal threshold (threshold of 0.1106) achieved an AUC of 0.938 [95% CI: 0.924–0.951] in identifying the primary outcome of OMI (*Figure 2*) on the overall test set. Model performance was comparable on both the EU internal (see Supplementary material online, *Figure S1A*) and US external testing data sets (see Supplementary material online, *Figure S1B*) and achieved an AUC of 0.946 (95% CI: 0.925–0.961) and of 0.903 (95% CI: 0.893–0.939), respectively (see Supplementary material online, *Figure S1*).

### Subgroup performance

**Figure 3:**
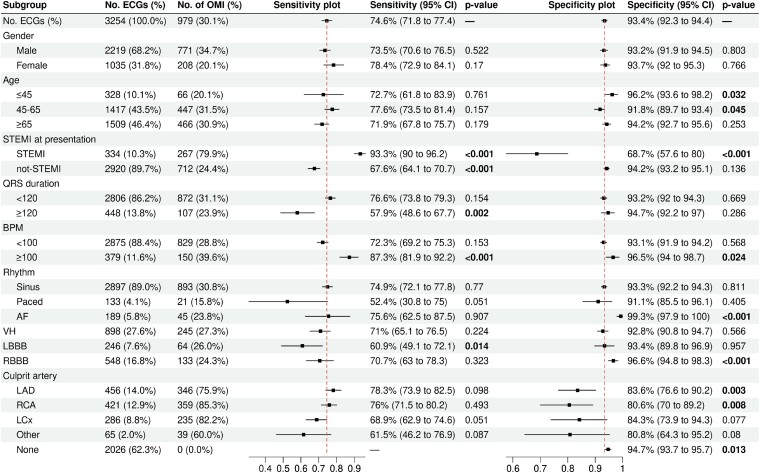
A subgroup analysis of the sensitivity and specificity of the occlusion myocardial infarction artificial intelligence model. The vertical dashed red line represents the overall artificial intelligence model sensitivity and specificity across all electrocardiograms in the testing data set. ECG, electrocardiogram; STEMI, ST-elevation myocardial infarction; AF, atrial fibrillation; VH, ventricular hypertrophy; LBBB, left bundle branch block; RBBB, right bundle branch block; LAD, left anterior descending artery; RCA, right coronary artery; LCx, left circumflex artery.

The average AI model performance of all individual ECGs in the testing data set was compared with different demographic and electrocardiographic subgroups (*Figure 3*). The model yielded stable sensitivities across gender and age groups (ranging from 71.9 to 78.4%). Specificity was slightly higher in patients under 45 (95.9%, *P* = 0.032) and in patients aged 45–65 (91.8%, *P* = 0.045). Sensitivity was higher for patients presenting with a STEMI ECG [93.3% (95% CI: 90.0–96.2%; *P* < 0.001) vs. 67.6% (95% CI: 64.1–70.7%; *P* < 0.001)], while specificity tended to be higher for patients presenting without STE on their index ECG [94.2% (95% CI: 93.2–94.3%), *P* = 0.136 vs. 68.7% (95% CI: 57.6–80.0%), *P* < 0.001]. Higher performance was recorded for ECGs with tachycardia over 100 b.p.m. [87.3% sensitivity (95% CI: 81.9–92.2%), *P* < 0.001 and 96.5% specificity (95% CI: 94.0–98.7%), *P* = 0.024], while the sensitivity of ECGs with broad QRS complex ≥120 ms was lower [57.9% sensitivity (95% CI: 48.6–67.7%), *P* = 0.002]. The performance of the model was consistent across ECG rhythms with a significantly higher specificity of 99.3% [(95% CI: 97.9–100%), *P* < 0.001] for ECGs with atrial fibrillation. Artificial intelligence model sensitivity did not significantly differ across different culprit artery territories; nevertheless, specificity was lower in patients with left anterior descending artery and right coronary artery culprit territories [83.6% (95% CI: 76.6–90.2%), *P* = 0.003 and 80.6% (95% CI: 70.0–89.2%), *P* = 0.008, respectively]. Model performance was comparable when tested on secondary definitions of OMI with different TIMI flow and troponin cut-off combinations, as well as the occurrence of percutaneous coronary intervention (PCI; *Table 3*).

**Table 3:** Performance of the occlusion myocardial infarction artificial intelligence model and analysis of different occlusion myocardial infarction outcome definitions across the grouped testing data sets (both Europe and USA)

OMI outcome definition	OMI AI model—optimal threshold^a^					
	Sens.	Spec.	PPV	NPV	AUC	MCC
Culprit TIMI 0–1	87.5% (83.4–91.3)	86.9% (85.4–88.4)	0.485 (0.445–0.528)	0.980 (0.973–0.987)	0.929 (0.912–0.944)	0.588 (0.548–0.628)
**Culprit TIMI 0–1 OR TIMI 2–3 with urgent PCI**	**80.6% (76.8–84.0)**	**93.7% (92.6–94.8)**	**0.780 (0.742–0.816)**	**0.946 (0.935–0.957)**	**0.938 (0.924–0.951)**	**0.735 (0.699–0.768)**
Culprit TIMI 0–1 OR TIMI 2–3 Trop T ≥500 ng/L	83.6% (79.9–86.9)	92.4% (91.1–93.6)	0.727 (0.688–0.765)	0.959 (0.949–0.968)	0.942 (0.928–0.955)	0.722 (0.685–0.756)
Culprit TIMI 0–1 OR TIMI 2–3 with Trop T ≥1000 ng/L	84.5% (80.8–88.0)	91.6% (90.3–92.8)	0.691 (0.65–0.73)	0.964 (0.955–0.972)	0.942 (0.928–0.955)	0.706 (0.667–0.74)
Culprit TIMI 0–2 OR TIMI 3 with Trop T ≥1000 ng/L and PCI performed	82.4% (78.6–86.0)	92.6% (91.3–93.7)	0.733 (0.694–0.771)	0.955 (0.945–0.965)	0.939 (0.925–0.952)	0.718 (0.68–0.753)

Bold values represent the primary outcome definition of OMI.

OMI, occlusion myocardial infarction; AI, artificial intelligence; STEMI, ST-elevation myocardial infarction; Sens., sensitivity; Spec., specificity; PPV, positive predictive value; NPV, negative predictive value; AUC, area under curve; MCC, Matthew’s correlation coefficient; TIMI, thrombolysis in myocardial infarction; Trop, troponin; PCI, percutaneous coronary intervention.

^a^Optimal threshold based on an ROC analysis (threshold of 0.1106).

### Artificial intelligence model benchmarking

The OMI AI model was compared against two standard criteria assessing the same 12-lead ECGs in the overall test set for the presence of OMI (*Table 4*). At the optimal threshold, the OMI AI model exhibited a significantly higher sensitivity in identifying OMI compared with STEMI criteria [80.6% (95% CI: 76.8–84.0%) vs. 32.5% (95% CI: 28.4–36.6%), *P* < 0.001] and was statistically equal to ECG experts [73.0% (95% CI: 68.7–77.0%)]. Accuracy in detecting OMI was equal between the OMI AI model and the experts and significantly higher when compared with STEMI criteria. Specificity was highest for STEMI criteria [97.7% (95% CI: 97.0–98.3%)] compared with ECG experts [95.7% (95% CI: 94.7–96.6%)] and OMI AI model [93.7% (95% CI: 92.6–94.8%)]. The comparison of all independently tested criteria for OMI diagnosis is summarized in Supplementary material online, *Table S1*.

**Table 4:** Head-to-head benchmark comparison in detecting the primary outcome definition of occlusion myocardial infarction

Comparator	Ref −	Ref +	Accuracy	Sensitivity	Specificity	PPV	NPV	AUC
OMI AI model—optimal threshold^a^								
−	1663	95	90.9% (89.7–92.0)	80.6% (76.8–84.0)	93.7% (92.6–94.8)	0.780 (0.742–0.816)	0.946 (0.935–0.957)	0.938 (0.924–0.951)
+	111	394						
STEMI criteria								
−	1733	330	83.6% (82.1–85.1)	32.5% (28.4–36.6)	97.7% (97.0–98.3)	0.795 (0.74–0.849)	0.840 (0.825–0.855)	0.651 (0.629–0.672)
+	41	159						
ECG experts								
−	1697	132	90.8% (89.5–91.9)	73.0% (68.7–77.0)	95.7% (94.7–96.6)	0.823 (0.785–0.857)	0.928 (0.916–0.94)	0.843 (0.821–0.864)
+	77	357						
OMI AI model—STEMI-matched specificity^b^								
−	1729	167	90.6% (89.4–91.8)	65.8% (61.3–70.0)	97.5% (96.7–98.1)	0.877 (0.841–0.908)	0.912 (0.899–0.925)	0.938 (0.924–0.951)
+	45	322						

Ref, reference; OMI, occlusion myocardial infarction; AI, artificial intelligence; STEMI, ST-elevation myocardial infarction; PPV, positive predictive value; NPV, negative predictive value; AUC, area under curve; ECG, electrocardiogram.

^a^Optimal threshold based on an ROC analysis (threshold of 0.1106).

^b^Threshold selected to match the specificity of STEMI criteria (threshold of 0.5995).

The mean time to OMI diagnosis was significantly shorter for the OMI AI model compared with STEMI criteria, 2.3 vs. 5.3 h, respectively (*P* < 0.001; see Supplementary material online, *Figure S2*), but comparable with ECG experts, with a mean time of 2.9 h (*P* = 0.08). Patients with OMI received interventions at a similar rate regardless of the presence of STEMI criteria and outcome definition [primary outcome definition, 97.3 vs. 95.9% (*P* = 0.570); strictest OMI outcome (TIMI 0–1 flow only), 96.3 vs. 92.4% (*P* = 0.358; see Supplementary material online, *Table S2*].

### Analysis of misclassified cases

Patients identified as OMI but who did not meet the primary outcome definition were labelled as OMI false positives; this occurred in 111 cases with the OMI AI model, in 41 cases with STEMI criteria, and in 77 cases by ECG experts (see Supplementary material online, *Table S3*). In OMI false positives with the AI model, the rate of myocardial injury (troponin elevation with absence of acute myocardial infarction) was significantly higher when compared with OMI false positives with STEMI criteria [16 (14.4%) vs. 1 (2.4%) respectively, *P* = 0.042] but similar to OMI false positives by ECG experts [11 (14.3%), *P* = 0.392].

Of the 330 OMI patients (67.5% of all OMI) missed by STEMI criteria (false negatives), only 112 (33.9%) had a time to revascularization of <2 h, while 133 of the remaining 218 false-negative OMI patients (61.0%) were correctly identified by the OMI AI model using the first ECG. These patients had a median revascularization time of 9.3 h (IQR 4.3, 16.9). The OMI AI model correctly classified 56 (42%) false negatives of ECG experts. These patients had a median time to CAG of 7.2 h (IQR 3.2, 17.2), and 58.9% had culprit lesions in the inferior or posterior territory.

## Discussion

We developed and validated a novel explainable AI model to detect acutely occluded or obstructive culprit coronary artery from a single individual 2.5 s 12-lead ECG recorded in patients with suspected ACS before cardiac catheterization. The model is superior to conventional STEMI criteria and comparable with interpretation by specialized ECG experts, blinded to all other clinical information, in detecting invasively confirmed acute coronary occlusion. High accuracy was upheld across two large, independent testing cohorts of ACS patients from Europe and USA, with robust performance across demographic, electrocardiographic, and infarct territory subgroups.

The present research is driven by the unmet need related to the suboptimal triage of ACS patients presenting with dynamic and often subtle ECG changes initially. Barely, 25% of patients with ACS present with typical ST-segment elevation on their initial ECG,^23^ and up to 35% of patients without such ST-segment elevation have total coronary occlusion discovered on delayed angiography.^24–28^ In addition, 20% of OMI patients meet STEMI criteria on the initial ECG, 30% on serial ECGs, and only 49% are recognized by cardiologists as STEMI.^29,30^ Compared with NSTEMI with a non-occlusive stenosis of the culprit coronary artery (NOMI),^2^ patients with OMI have far higher mortality and worse left ventricular function, in spite of presenting at a younger age and with fewer comorbidities.^4^

Several previous studies deployed machine learning to triage patients presenting with ACS, however, bearing multiple limitations.^23,31–43^ The majority of these studies did not validate the occlusive or flow-limiting culprit lesions on coronary angiogram and relied on a subjective majority vote of board-certified cardiologists interpreting the ECG with STEMI as the surrogate for OMI.^23,32–36^ In addition, they often employed a spectrum of input clinical features in addition to the ECG waveform restricting their practical, real-world implementation.^16,37–43^ Moreover, they depended on the acquisition of digital 10 s ECGs from a single vendor limiting the broader adoption.^23,31–43^ Finally, their validation was not scrutinized in sizeable external and international data sets.

Our study is characterized by several methodological strengths. First, the OMI AI model is trained using deep learning methodology on an international cohort of standardized 12-lead ECG waveforms from multiple vendors. Second, the OMI reference standard used for model development and evaluation was acute occlusive culprit stenosis confirmed angiographically. Third, the AI model interprets OMI using only ECG waveforms as input, independent of patient demographics or further clinical information. Using this robust methodology, the OMI AI model achieved superior accuracy within an independent cohort. Likewise, the AI model demonstrated sustained high performance (>0.92 AUC) on both EU internal testing data sets with the natural prevalence of OMI within a cohort of ACS patients and an external validation set of patients from two independent US centres. The OMI AI model yielded a statistically superior performance to STEMI criteria and equal performance to ECG experts when compared using six complementary performance metrics. More specifically, the model outperformed standard ECG millimetre criteria in detecting acute coronary occlusion offering an over two-fold increase in sensitivity while maintaining high specificity comparable to STEMI criteria. The presented OMI AI model detects OMI significantly earlier (by 3 h) compared with current guideline-recommended criteria. The performance of the model has been retained across coronary vascular territories displaying high specificity in complex clinical settings such as atrial fibrillation or tachycardia. This could be attributed to the AI model’s deep learning ability to identify new ECG patterns.

### Clinical implications

**Figure 4:**
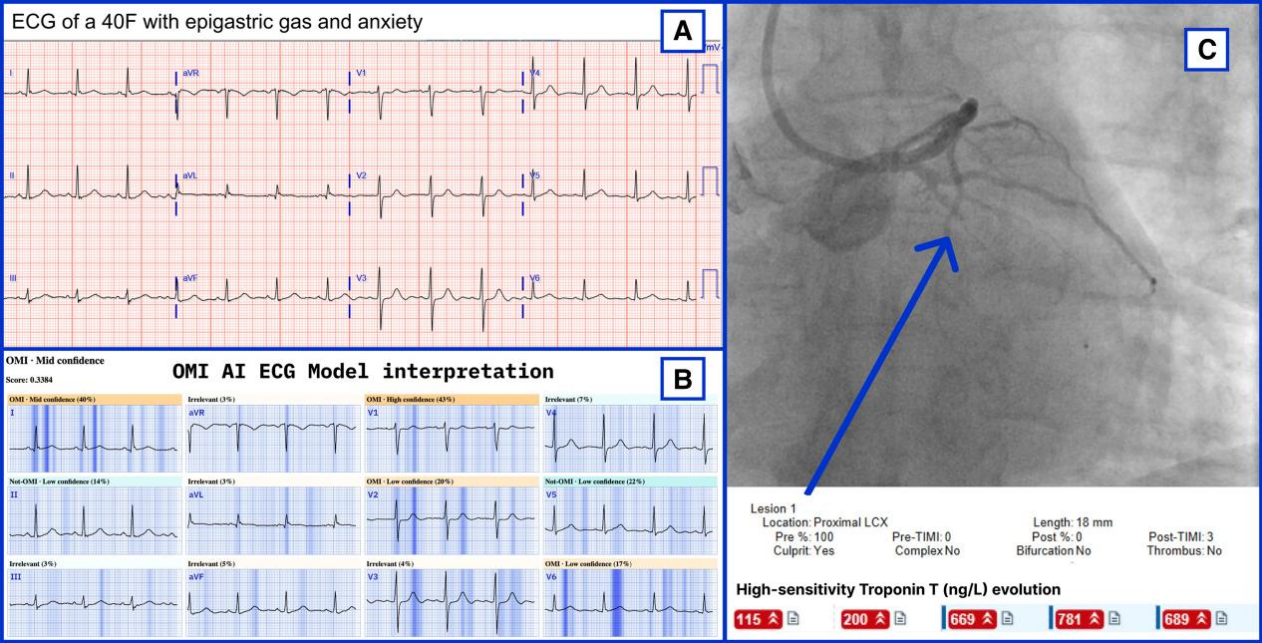
A real-world demonstration of an occlusion myocardial infarction artificial intelligence true-positive electrocardiogram downloaded from Twitter. (*A*) The original electrocardiogram posted to Twitter by Brooks Walsh, MD (https://twitter.com/BrooksWalsh, emergency physician at the Bridgeport Hospital, Bridgeport, CT, USA) with the occlusion myocardial infarction artificial intelligence model interpretation (above the optimal threshold); (*B*) the occlusion myocardial infarction artificial intelligence electrocardiogram model interpretation (above optimal threshold) with model explainability; (*C*) the angiogram of the occluded proximal left circumflex culprit artery and high-sensitivity troponin T evolution for this case.

**Figure 5:**
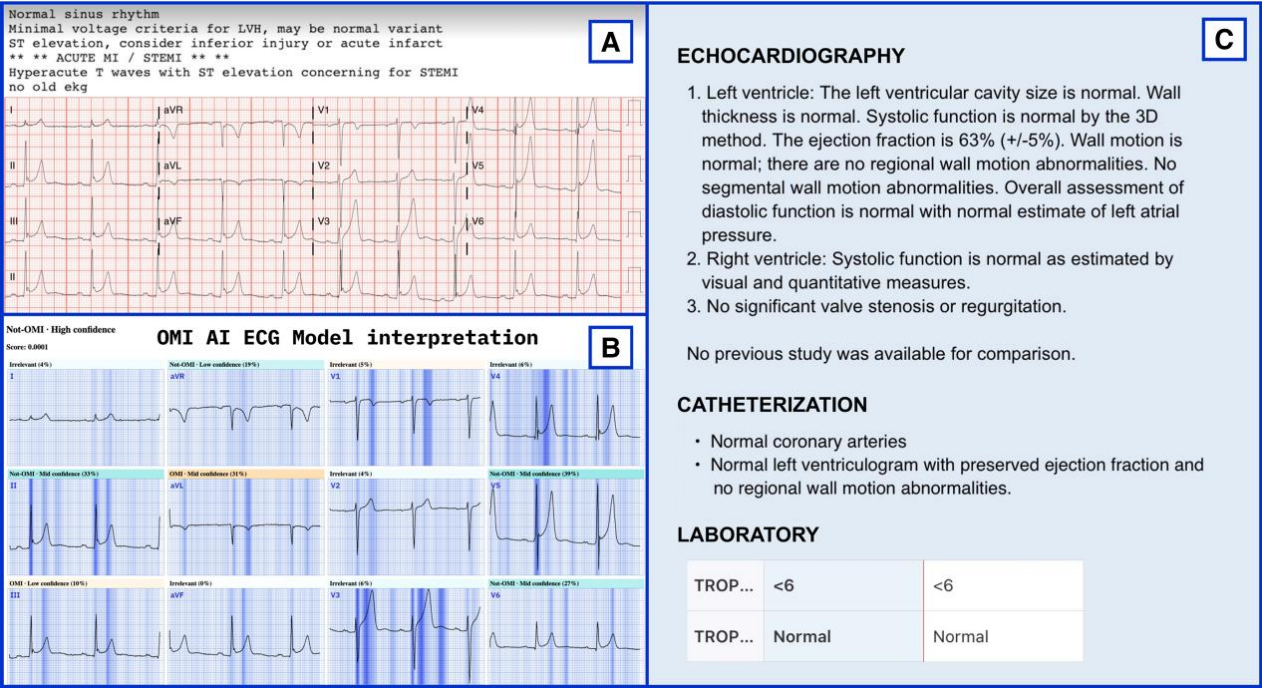
A real-world demonstration of occlusion myocardial infarction artificial intelligence true-negative electrocardiogram downloaded from Twitter. (*A*) The original electrocardiogram posted to Twitter by Pendell Meyers, MD (https://twitter.com/PendellM, emergency physician at the Carolinas Medical Centre, Charlotte, NC, USA). Both the automated diagnostic statements and the attending physician misinterpreted this electrocardiogram, subsequently triggering a false-positive ST-elevation myocardial infarction cathlab activation; (*B*) the automatically digitized electrocardiogram with a very low occlusion myocardial infarction artificial intelligence model output (below the optimal threshold) and model explainability; (*C*) the echocardiography, catheterization, and laboratory report for this case.

This study has several implications for the future management of ACS. The OMI AI model paired with digitization technology offers an accurate detection of patients with OMI using single-standard 12-lead ECG tracings independent of the ECG vendor or its format (*Figures 4* and *5* show real-world demonstration). Specifically, such accurate and timely ECG-based ACS diagnosis at the time of first patient contact could prompt a swift coronary intervention as recommended currently in the case of standard STEMI criteria. The rapid reperfusion in such management can consequently limit the burden of myocardial injury with favourable impact on clinical outcomes. In this regard, the model reliably detected OMI on average 3 h earlier than the current guideline-based ECG standards suggesting its potential to streamline the timely referral of ACS patients at risk for poor outcomes.

### Limitations

Several limitations are to be considered. Although validated in multi-centre, international cohorts of patients, our study lacks prospective validation. In clinical practice, the decision to refer for early angiography in patients presenting with NSTEMI, as well as to treat by revascularization or conservatively, is based not only on ECG but often encompasses additional clinical criteria. Nevertheless, our results show less than half (43.9%) of OMI patients undetected by standard STEMI criteria that could have had accelerated access to PCI based on the AI model detection truly underwent revascularization within 2 h. However, their median time to revascularization was delayed by over 9 h. There were significant differences in clinical presentation and management between patients in the Europe and USA due to variations in the standard of care. Although the model has demonstrated robust performance across various subgroups, its sensitivity was lower in patients with left bundle branch block and broad QRS morphology. The outcome of OMI relied on a visual verification of TIMI flow on angiograms, which may be subjective when compared with TIMI frame counting, and was not performed in an independent core lab. Culprit lesions with TIMI 2/3 flow requiring urgent revascularization were encompassed in the primary outcome since up to one-fourth of STEMI patients have pharmacological or spontaneous reperfusion at the time of angiography. In this regard, we present an AI model performance, utilizing broad ranges of peak troponin cut-offs, which may serve as more appropriate indicators of significant myocardial infarction resulting from these lesions. The OMI AI model detects OMI with a binary granularity. It is understood that the different stages of culprit coronary lesion leading to ACS, in terms of dynamics (active or reperfused) and time (acute or subacute), can have an influence on patient outcomes and the timing of invasive strategies. Lastly, our study is not generalizable to a broader population of asymptomatic patients and was not designed to quantify other relevant clinical endpoints such as mortality, in-hospital complications, or major adverse cardiovascular events (MACE). Future work should address these limitations and observe the AI model efficacy and clinical benefit deployed in a prospective cohort of ACS patients.

## Conclusions

We have developed and validated an OMI AI model that is able to accurately detect ACS patients with the angiographically confirmed occlusion of culprit coronary arteries using only single-standard 12-lead ECGs in a large international, multi-centre cohort of ACS patients. Our AI model outperformed gold-standard STEMI criteria in the diagnosis of OMI, but further prospective clinical studies are needed to define the role of the OMI AI model in guiding ACS triage and the timely referral of patients benefiting from immediate revascularization.

## Supplementary material

Supplementary material is available at *European Heart Journal – Digital Health*.

## Supplementary Material

ztad074_Supplementary_Data

## Data Availability

The OMI AI ECG model is available for external validation, benchmarking, and research use at: https://bit.ly/omi-ai-ecg. The data set is not available for public sharing, given our institutional review board approval restrictions.
