# Supplementary material for: International evaluation of an artificial intelligence–powered electrocardiogram model detecting acute coronary occlusion myocardial infarction
Source: Eur Heart J Digit Health. 2023 Nov 28;5(2):123–33. doi: 10.1093/ehjdh/ztad074 (PMC10944682; doi:10.1093/ehjdh/ztad074)
Supplement: ztad074_Supplementary_Data [file ztad074_supplementary_data.docx]

**INTERNATIONAL EVALUATION OF AN ARTIFICIAL INTELLIGENCE-POWERED ECG MODEL DETECTING ACUTE CORONARY OCCLUSION MYOCARDIAL INFARCTION**

*SUPPLEMENTARY MATERIAL*

Robert Herman MD^1,2,3^, H. Pendell Meyers MD^4^, Stephen W. Smith MD^5,6^, Dario T. Bertolone MD^1,2^, Attilio Leone MD^1,2^, Konstantinos Bermpeis MD^1,2^, Michele M. Viscusi MD^1,2^, Marta Belmonte MD^1,2^, Anthony Demolder MD, PhD^3^, Vladimir Boza MSc, PhD^3,7^, Boris Vavrik MSc^3^, Viera Kresnakova MSc, PhD^3,8^, Andrej Iring MSc^3^, Michal Martonak MSc^3^, Jakub Bahyl MSc^3^, Timea Kisova BSc^3,9^, Dan Schelfaut, MD^2^, Marc Vanderheyden, MD^2^, Leor Perl, MD^10^, Emre K. Aslanger, MD^11^, Robert Hatala, MD, PhD^12^, Wojtek Wojakowski MD, PhD^13^, Jozef Bartunek MD, PhD^2^, Emanuele Barbato MD, PhD^14^

^1^Department of Advanced Biomedical Sciences, University of Naples Federico II, Naples, Italy; ^2^Cardiovascular Centre Aalst, Aalst, Belgium; ^3^Powerful Medical, Bratislava, Slovakia; ^4^Department of Emergency Medicine, Carolinas Medical Center, North Carolina, USA; ^5^Department of Emergency Medicine, University of Minnesota, Minneapolis, Minnesota, USA; ^6^Department of Emergency Medicine, Hennepin Healthcare, Minneapolis, Minnesota, USA; ^7^Faculty of Mathematics, Physics and Informatics, Comenius University in Bratislava, Bratislava, Slovakia; ^8^Department of Cybernetics and Artificial Intelligence, Technical University of Kosice, Kosice, Slovakia; ^9^Barts and The London School of Medicine and Dentistry, London, United Kingdom; ^10^Department of Cardiology, Rabin Medical Center, Petah Tikvah, Israel; ^11^Department of Cardiology, Basaksehir Cam and Sakura City Hospital, Istanbul, Turkey, ^12^Department of Arrhythmia and Pacing, National Institute of Cardiovascular Diseases, Bratislava, Slovakia; ^13^Department of Cardiology and Structural Heart Diseases, Medical University of Silesia, Katowice, Poland, ^14^Department of Clinical and Molecular Medicine, Faculty of Medicine and Psychology, Sapienza University of Rome, Italy.

Address for correspondence:

**Robert Herman, MD**

Cardiovascular Centre,

OLV Hospital,

Moorselbaan 164, 9300 Aalst, Belgium

Phone: +43 676 374 6517

Email: robi.herman@gmail.com

Supplemental Table 1. Summary of benchmark comparison across industry-standard performance metrics. The benchmark summary compares metrics and confidence intervals for each combination of OMI detection.

(+) denotes a significantly better performance for that specific metric; (-) denotes a significantly worse performance for that specific metric; (0) denotes a statistically equal performance (confidence intervals overlap) for that specific metric.

| **Benchmark comparison** | **Sens.** | **Spec.** | **PPV** | **NPV** | **MCC** | **AUC** | **Adjudication** |
| --- | --- | --- | --- | --- | --- | --- | --- |
| **OMI AI Model^±^** vs. STEMI Criteria | + | - | - | + | + | + | Significantly better |
| **OMI AI Model^±^** vs. ECG Experts | + | - | - | + | 0 | + | Equal |
| ECG Experts vs STEMI Criteria | + | - | 0 | + | + | + | Significantly better |
| **±** Optimal threshold based on ROC analysis (threshold of 0.1106)  OMI, Occlusion myocardial infarction; AI, artificial intelligence; STEMI, ST-elevation myocardial infarction; Sens., Sensitivity; Spec., Specificity; PPV, Positive predictive value; NPV, Negative predictive value; AUC, Area under curve; MCC, Matthews correlation coefficient. | | | | | | | |

Supplemental Table 2. Index contact interventions of patients stratified according to OMI outcome and presence of STEMI criteria.

| **Parameter** | **Category** | **Primary OMI outcome^±^** | | | **Strictest OMI outcome**^‡^ | | |
| --- | --- | --- | --- | --- | --- | --- | --- |
|  |  | *STEMI*  *(n=146)* | *Not-STEMI*  *(n=343)* | *P-value* | *STEMI*  *(n=109)* | *Not-STEMI*  *(n=171)* | *P-value* |
| Culprit Identified, n (%) | Native vessel | 145 (99.3) | 326 (95.0) | **0.042** | 108 (99.1) | 164 (95.9) | 0.156 |
| Culprit Identified, n (%) | Graft vessel | 1 (0.7) | 17 (5.0) | **0.042** | 1 (0.9) | 7 (4.1) | 0.156 |
| Treatment, n (%) | Conservative | 4 (2.7) | 12 (3.5) | 0.591 | 4 (3.7) | 12 (7.0) | 0.358 |
| Treatment, n (%) | PCI | 142 (97.3) | 329 (95.9) | 0.591 | 105 (96.3) | 158 (92.4) | 0.358 |
| ± Culprit TIMI 0-2 OR TIMI 3 with Trop T ≥1000ng/L  ‡ Culprit TIMI 0-1  OMI, Occlusion myocardial infarction; STEMI, ST-elevation myocardial infarction; TIMI, Thrombolysis In Myocardial Infarction; Trop, Troponin; PCI, percutaneous coronary intervention. | | | | | | | |

| **Parameter** | ***Category*** | **False positives** | | | **P-value** |
| --- | --- | --- | --- | --- | --- |
|  |  | *OMI AI Model (n=111)* | *STEMI criteria (n=41)* | *ECG Experts (n=77)* |  |
| Peak Troponin T (ng/L), median [Q1,Q3] |  | 155.0 [26.7,843.5] | 21.5 [2.1,435.0] | 150.0 [22.6,995.8] | 0.085 |
| Time to CAG (hours), median [Q1,Q3] |  | 8.3 [1.4,18.7] | 4.4 [0.9,13.5] | 3.8 [1.0,18.3] | 0.297 |
| Time to Revascularization (hours), median [Q1,Q3] |  | 15.8 [1.9,21.5] | 0.4 [0.3,0.9] | 1.6 [0.5,3.2] | **0.006** |
| Culprit Identified, n (%) | *None* | 86 (77.5) | 38 (92.7) | 60 (77.9) | 0.223 |
|  | *Native vessel* | 24 (21.6) | 3 (7.3) | 17 (22.1) | 0.223 |
|  | *Graft vessel* | 1 (0.9) |  |  | 0.223 |
| TIMI Flow, n (%) | *TIMI-0* | 0 (0.0) | 0 (0.0) | 1 (5.6) | 0.353 |
|  | *TIMI-1* | 1 (3.8) | 0 (0.0) | 1 (5.6) | 0.353 |
|  | *TIMI-2* | 2 (7.7) | 0 (0.0) | 5 (27.8) | 0.353 |
|  | *TIMI-3* | 22 (88.0) | 3 (100.0) | 10 (58.8) | 0.353 |
| Culprit artery, n (%) | *LMCA* | 2 (8.0) |  | 2 (11.8) | 0.965 |
|  | *LAD* | 10 (40.0) | 2 (66.7) | 6 (35.3) | 0.965 |
|  | *LCx* | 4 (16.0) |  | 3 (17.6) | 0.965 |
|  | *RCA* | 8 (32.0) | 1 (33.3) | 6 (35.3) | 0.965 |
| Vessel Stenosis (%), median [Q1,Q3] |  | 90.0 [60.0,95.0] | 65.0 [40.0,95.0] | 90.0 [75.0,99.0] | 0.469 |
| Treatment, n (%) | *Conservative* | 63 (70.0) | 14 (82.4) | 45 (72.6) | 0.579 |
|  | *PCI* | 27 (30.0) | 3 (17.6) | 17 (27.4) | 0.579 |
| Myocardial injury, n (%) |  | 51 (45.9) | 12 (29.3) | 38 (49.4) | 0.096 |
| Myocardial injury and PCI, n (%) |  | 16 (14.4) | 1 (2.4) | 11 (14.3) | **0.042** |
| *Significant difference between OMI AI model and STEMI criteria  Cat., category; EU, Europe; US, United States; CAG, coronary angiography; ECG, electrocardiogram; STEMI, ST-elevation myocardial infarction; OMI, Occlusion myocardial infarction; AI, artificial intelligence; SD, standard deviation; LMCA, left main coronary artery; LAD, left anterior descending artery; LCx, left circumflex artery; RCA, right coronary artery; PDA, posterior descending artery; RI, ramus interventricularis; TIMI, Thrombolysis In Myocardial Infarction; PCI, percutaneous coronary intervention. | | | | | |

Supplemental Table 3. Baseline and procedural characteristics of false positive contacts.

Supplemental Table 4. Baseline and procedural characteristics of false negative contacts.

| **Parameter** | ***Category*** | **False negatives** | | | **P-value** |
| --- | --- | --- | --- | --- | --- |
|  |  | *OMI AI Model (n=95)* | *STEMI criteria (n=330)* | *ECG Experts (n=132)* |  |
| Peak Troponin T (ng/L), median [Q1,Q3] |  | 580.0 [176.8,1730.0] | 1496.5 [489.4,3987.5] | 810.0 [189.7,1730.0] | **<0.001** |
| Time to CAG (hours), median [Q1,Q3] |  | 8.3 [3.1,17.6] | 4.2 [1.5,13.4] | 8.1 [3.3,17.7] | **<0.001** |
| Time to Revascularization (hours), median [Q1,Q3] |  | 8.3 [3.2,17.8] | 4.1 [1.6,13.4] | 8.3 [3.5,18.3] | **<0.001** |
| Culprit Identified, n (%) | *None* |  |  |  | 0.211 |
|  | *Native vessel* | 88 (92.6) | 314 (95.2) | 120 (90.9) | 0.211 |
|  | *Graft vessel* | 7 (7.4) | 16 (4.8) | 12 (9.1) | 0.211 |
| TIMI Flow, n (%) | *TIMI-0* | 29 (30.5) | 142 (43.0) | 36 (27.3) | **0.036** |
|  | *TIMI-1* | 6 (6.3) | 22 (6.7) | 8 (6.1) | **0.036** |
|  | *TIMI-2* | 13 (13.7) | 42 (12.7) | 21 (15.9) | **0.036** |
|  | *TIMI-3* | 47 (49.5) | 124 (37.6) | 67 (50.8) | **0.036** |
| Culprit artery, n (%) | *LMCA* | 1 (1.1) | 3 (0.9) | 3 (2.3) | 0.86 |
|  | *LAD* | 29 (30.5) | 121 (36.7) | 39 (29.5) | 0.86 |
|  | *LCx* | 27 (28.4) | 83 (25.2) | 43 (32.6) | 0.86 |
|  | *RCA* | 33 (34.7) | 111 (33.6) | 42 (31.8) | 0.86 |
| Vessel Stenosis (%), median [Q1,Q3] |  | 95.0 [90.0,100.0] | 99.0 [90.0,100.0] | 95.0 [90.0,100.0] | **0.003** |
| Collateral flow, n (%) | *NONE* | 53 (88.3) | 163 (88.6) | 78 (85.7) | 0.949 |
|  | *MILD* | 1 (1.7) | 7 (3.8) | 4 (4.4) | 0.949 |
|  | *MODERATE* | 5 (8.3) | 12 (6.5) | 7 (7.7) | 0.949 |
|  | *HIGH* | 1 (1.7) | 2 (1.1) | 2 (2.2) | 0.949 |
| Treatment, n (%) | *Conservative* | 4 (4.2) | 12 (3.6) | 4 (3.0) | 0.712 |
|  | *PCI* | 91 (95.8) | 316 (95.8) | 126 (95.5) | 0.712 |
| Myocardial injury, n (%) |  | 95 (100.0) | 330 (100.0) | 132 (100.0) | 1 |
| Myocardial injury and PCI, n (%) |  | 91 (95.8) | 316 (95.8) | 126 (95.5) | 0.988 |
| *Significant difference between OMI AI model and STEMI criteria  Cat., category; EU, Europe; US, United States; CAG, coronary angiography; ECG, electrocardiogram; STEMI, ST-elevation myocardial infarction; OMI, Occlusion myocardial infarction; AI, artificial intelligence; SD, standard deviation; LMCA, left main coronary artery; LAD, left anterior descending artery; LCx, left circumflex artery; RCA, right coronary artery; PDA, posterior descending artery; RI, ramus interventricularis; TIMI, Thrombolysis In Myocardial Infarction; PCI, percutaneous coronary intervention. | | | | | |

Supplemental Table 5. Performance of OMI AI Model with STEMI criteria matched specificity and analysis of different OMI outcome definitions across the grouped testing datasets (both EU and US). *In bold primary outcome definition of OMI.*

| **OMI outcome definition** | **OMI AI Model –**  **STEMI matched specificity^‡^** | | | | | |
| --- | --- | --- | --- | --- | --- | --- |
|  | *Sens.* | *Spec.* | *PPV* | *NPV* | *AUC* | *MCC* |
| Culprit TIMI 0-1 | 78.9% (73.9-83.5) | 92.6% (91.4-93.7) | 0.602 (0.552-0.651) | 0.969 (0.961-0.976) | 0.929 (0.912-0.944) | 0.639 (0.593-0.681) |
| **Culprit TIMI 0-1 *OR* TIMI 2-3 with Urgent PCI** | 65.8% (61.3-69.9) | 97.5% (96.7-98.1) | 0.877 (0.841-0.909) | 0.912 (0.899-0.925) | 0.938 (0.924-0.951) | 0.707 (0.667-0.739) |
| Culprit TIMI 0-1 *OR* TIMI 2-3 Trop T ≥500 ng/L | 71.1% (66.4-75.2) | 97.0% (96.1-97.7) | 0.850 (0.811-0.884) | 0.933 (0.921-0.944) | 0.942 (0.928-0.955) | 0.730 (0.691-0.767) |
| Culprit TIMI 0-1 *OR* TIMI 2-3 with Trop T ≥1000 ng/L | 73.4% (68.8-77.5) | 96.5% (95.6-97.3) | 0.826 (0.785-0.861) | 0.942 (0.931-0.952) | 0.942 (0.928-0.955) | 0.733 (0.692-0.768) |
| Culprit TIMI 0-2 *OR* TIMI 3 with Trop T ≥1000 ng/L and PCI performed | 69.5% (64.8-73.6) | 97.0% (96.1-97.7) | 0.850 (0.81-0.885) | 0.928 (0.916-0.939) | 0.939 (0.925-0.952) | 0.719 (0.679-0.756) |
| ‡ Threshold selected to match specificity of STEMI criteria (threshold of 0.5995)  OMI, Occlusion myocardial infarction; AI, artificial intelligence; STEMI, ST-elevation myocardial infarction; Sens., Sensitivity; Spec., Specificity; PPV, positive predictive value; NPV, Negative predictive value; AUC, Area under curve; MCC, Matthews correlation coefficient; TIMI, Thrombolysis In Myocardial Infarction; Trop, Troponin; PCI, percutaneous coronary intervention. | | | | | | |





Supplemental Figure 1. AI model performance on EU and US testing cohorts separated. *Figure shows the ROC curve of OMI AI Model (red) and sensitivity and specificity of OMI AI Model Optimal threshold (red X), STEMI criteria (green dot) and ECG experts (purple cross). Panel A shows the AUC of the OMI AI Model of 0.946 (n=1,630 contacts [15.95% OMI] on the EU internal testing cohort; Panel B shows the AUC of the OMI AI Model of 0.903 (n=633 contacts [36.18% OMI] on the US external testing cohort.* ROC, Receiver operating curve; EU, Europe; US, United States; OMI, Occlusion myocardial infarction; AI, artificial intelligence; STEMI, ST-elevation Myocardial Infarction; ECG, electrocardiogram; Sens, Sensitivity; Spec, Specificity; AUC, Area under the curve.


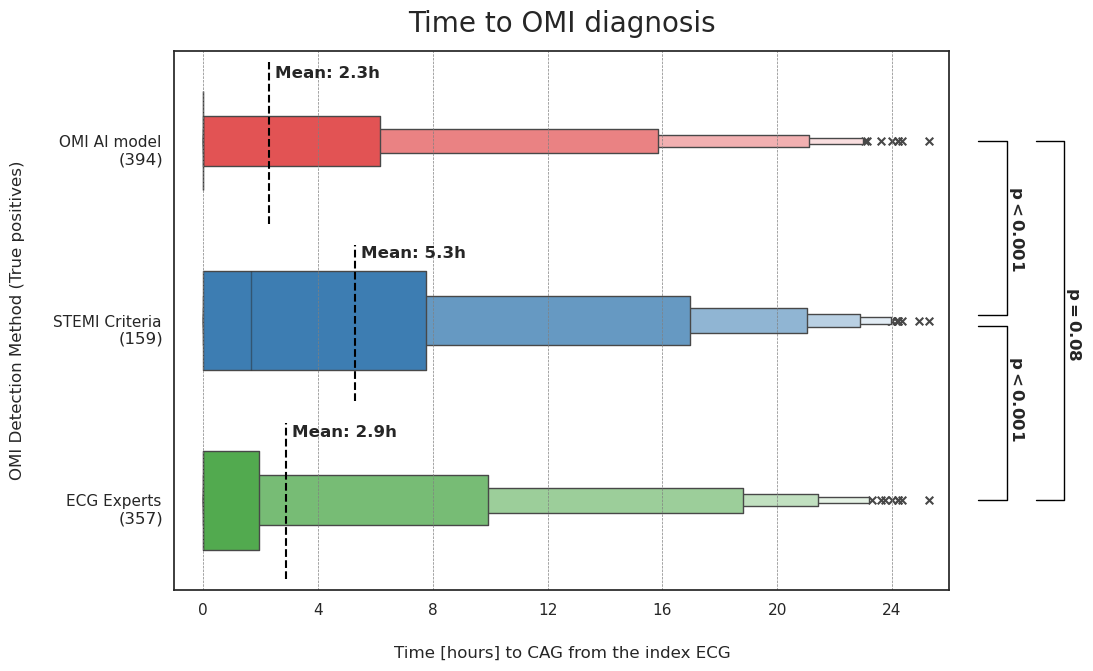


Supplemental Figure 2. Time to OMI diagnosis by detection method. *Graph shows the time to OMI diagnoses (primary outcome definition) when relying on individual detection methods. If the detection method detected OMI on the first ECG, the time to diagnosis is 0. If the detection method did not catch OMI on any ECG, the time to diagnosis corresponds to the time to coronary angiography.*

**
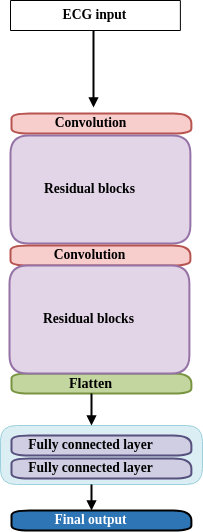
**

Supplemental Figure 3. OMI AI ECG deep neural network model architecture.

**SUPPLEMENTAL METHODS**

**Online ACS database description.** The online ACS database used for model development is an anonymized repository of publicly available cases collected by the co-authors Stephen Smith and Pendell Meyers. Microsoft Excel software was employed to gather ECG images, expert interpretations, and invasive outcomes (where applicable). Proprietary ECG digitization technology (Powerful Medical, Samorin, Slovakia) was used to digitize the ECG images in the database. It is important to note that ECGs from this database were solely utilized for model development and were not incorporated into the test sets.

**AI model explainability.** We have incorporated two novel explainability features inspired by the Local Interpretable Model-agnostic Explanations (LIME) method^1^ to enhance interpretability of the OMI AI ECG model: 1) lead specific diagnosis and influence and 2) time frame relevance. Both explainability features involve making minor modifications to the ECG, observing the changes in the prediction, and subsequently establishing a linear model centered around the presented ECG. The calculation of per lead influence involved determining the alteration in prediction following the insertion of each individual lead to the ECG. The magnitude of this change served as an assessment of the relevance in each lead. For per time frame relevance, a 25ms window in each lead was selected to compute the corresponding shift in the window that would impact the ECG prediction. To simplify visualization, only the magnitude of the change was plotted.

# REFERENCES

1. Ribeiro MT, Singh S, Guestrin C. " Why should i trust you?" Explaining the predictions of any classifier. In: *Proceedings of the 22nd ACM SIGKDD international conference on knowledge discovery and data mining*. *2016*, p. 1135-1144.
